# High temperature suppressed SSC self-renewal through S phase cell cycle arrest but not apoptosis

**DOI:** 10.1186/s13287-019-1335-5

**Published:** 2019-07-29

**Authors:** Jia Wang, Wei-Jun Gao, Shou-Long Deng, Xiang Liu, Hua Jia, Wen-Zhi Ma

**Affiliations:** 1 Key Laboratory of Fertility Preservation and Maintenance of Ministry of Education, and Key Laboratory of Reproduction and Genetics of Ningxia Hui Autonomous Region, Department of Anatomy, Histology and Embryology, School of Basic Medical Science, Ningxia Medical University, Yinchuan, 750004 China; 2 CAS Key Laboratory of Genome Sciences and Information, Beijing Institute of Genomics, Chinese Academy of Sciences, Beijing, 100101 China; 3 Center for Reproductive Biology and Health, College of Agricultural Sciences, The Pennsylvania State University, University Park, PA 16802 USA

**Keywords:** High temperature, SSCs, Self-renewal, Cell cycle arrest, Apoptosis

## Abstract

**Background:**

High temperature has a very adverse effect on mammalian spermatogenesis and eventually leads to sub- or infertility through either apoptosis or DNA damage. However, the direct effects of heat stress on the development of spermatogonial stem cells (SSCs) are still unknown because SSCs are rare in the testes.

**Methods:**

In the present study, we first used in vitro-cultured SSCs to study the effect of heat shock treatment on SSC development. Then, we used RNA-Seq analysis to identify new genes or signalling pathways implicated in the heat stress response.

**Results:**

We found that 45 min of 43 °C heat shock treatment significantly inhibited the proliferation of SSCs 2 h after treatment but did not lead to apoptosis. In total, 17,822 genes were identified by RNA-Seq after SSC heat shock treatment. Among these genes, we found that 200 of them had significantly changed expression, with 173 upregulated and 27 downregulated genes. The number of differentially expressed genes in environmental information processing pathways was 37, which was the largest number. We screened the candidate JAK-STAT signalling pathway on the basis of inhibition of cell cycle progression and found that the JAK-STAT pathway was inhibited after heat shock treatment. The flow cytometry results further confirmed that heat stress caused S phase cycle arrest of SSCs.

**Conclusion:**

Our results showed that heat shock treatment at 43 °C for 45 min significantly inhibited SSC self-renewal through S phase cell cycle arrest but not apoptosis.

## Background

Spermatogenesis is a process by which spermatogonial stem cells (SSCs) self-renew and differentiate into sperm. Any error during spermatogenesis results in male infertility. Infertility occurs in 10–15% of all couples, and male factors account for 50% of cases. High temperature is one of the causes of male infertility [1]. Cryptorchidism or increased scrotal temperature leads to nonobstructive azoospermia or asthenozoospermia.

The scrotum is generally 2–7 °C cooler than the core body temperature in most male mammals, and the temperature of the testes is tightly regulated by a heat exchange system [2]. If the testes fail to descend into the scrotum during postnatal development, they are exposed to elevated temperature (the core body temperature) and lose germ cells [3]. Male germ cells (especially haploid spermatids) are significantly reduced or completely lost in the cryptorchid testes [4]. The transition of gonocytes into type A dark spermatogonia (SSCs) in cryptorchid testes is impaired [5]. Thus, the thermoregulation of the testes is essential for spermatogenesis. The reason why most mammals have evolved to maintain their testes at low temperatures remains unclear [6].

Scrotal high temperatures led to the interruption of spermatogenesis and reductions in sperm quality and quantity [7, 8]. Many experiments have shown that a single heat stress treatment of the testicles can induce apoptosis of heat-susceptible germ cells (late pachytene and diplotene spermatocytes and early round spermatids), resulting in the interruption of spermatogenesis and reversible transient sterility or DNA damage even if the germ cells escaped apoptosis. p53-dependent or p53-independent pathways and p38 mitogen-activated protein kinase (MAPK) upstream signal activation are involved in mitochondria-mediated apoptosis of spermatogenic cells [9, 10]. The apoptosis of spermatogenic cells causes depletion of spermatogenic cells in the seminiferous tubules, resulting in hollow tubules, spermatogenesis interruption and infertility. In addition, high temperatures can also cause sperm DNA breakage, oxidative damage and downregulation of protamine expression, which result in the destruction of sperm DNA integrity [11]. High temperature stress or varicocele increases the production of reactive oxygen species (ROS) in the testicles, and the excessive ROS attack lipoproteins and unsaturated fatty acids and damage proteins and DNA [12, 13]. In general, the lack of superoxide dismutase (SOD) in the testicles makes spermatogenic cells more sensitive than other cells to heat stress [12, 14–16]. The sperm in the epididymis is also affected by heat, which leads to sperm DNA damage that might result in subfertility of affected males or offspring deformity [17]. Spermatogonia are heat-tolerant germ cells. A study showed that stress granules are formed in spermatogonia after heat stress and confer resistance to apoptosis by suppressing the p38 MAPK pathway [18]. However, the direct effects of increased scrotal temperature on SSCs are still unknown because the number of SSCs accounts for as few as 0.03% of total adult testis cells [19].

In the present study, to provide insight into how heat shock treatment regulates the behaviour of SSCs, we first used in vitro-cultured SSCs to study the effects of heat shock treatment on SSC proliferation and apoptosis. Then, we used RNA-Seq analysis to identify new genes or signalling pathways implicated in the heat stress response.

## Materials and methods

### SSC culture

The CD1 SSC cell line from mice was donated by professor Wu Ji’s laboratory from Shanghai Jiao Tong University. The culture medium was based on Minimum Essential Medium α (MEM-α) (12571-063, Gibco, Grand Island, NY, USA) containing 2 mM glutamine (G7012, Sigma, MO, USA), 10% foetal bovine serum (FBS) (16000-36, Gibco), 0.5× pen/strep (15240-062, Invitrogen, Grand Island, NY, USA), 1× nonessential amino acid (NEAA) (11140-050, Gibco) solution, 1× β-mercaptoethanol (β-ME) (M3148, Sigma), 25 μg/ml insulin (I1882, Sigma), 100 μg/ml transferrin (T1428, Sigma), 60 μM putrescine (P5780, Sigma), 60 ng/ml progesterone (P8783, Sigma), 40 ng/ml glial cell line-derived neurotrophic factor (GDNF) (512-GF-050, R&D Systems, Minneapolis, MN, USA) and 3–5 ng/ml basic fibroblast growth factor (bFGF) (F0291, Sigma). The feeder layer cells were STO cells treated with mitomycin (M0503, Sigma). The SSCs were incubated at 37 °C in the presence of 5% CO_2_.

### Heat shock treatment

The SSCs were divided into a control group and a heat shock-treated group. We seeded 1.6 × 10^4^ cells per well into 6-cell plates or 2 × 10^3^ cells per well into 96-cell plates, with six parallel wells for each group. The cells in the heat shock-treated group were cultured in a 5% CO_2_ incubator at a constant temperature of 43 °C for 10 min, 15 min, 30 min, 45 min or 60 min and then transferred to another 5% CO_2_ incubator at 37 °C. The cells in the control group were cultured in a 5% CO_2_ incubator at 37 °C.

### Proliferation assay

A CCK-8 kit (MAC218, Meilunbio, Dalian, China) was used to detect SSC proliferation in the control and heat shock-treated groups. CCK-8 reagent (10 μl) was added to the medium (100 μl) in each well 2 h and 18 h after the heat shock treatment. After incubation for 2 h, the optical density value (OD value) was measured at 450 nm using a microplate reader (Multiskan GO, Thermo Fisher Scientific, Rockford, IL, USA). A growth curve was drawn based on the mean value of the eight counts in each group.

### Immunofluorescence staining

SSCs from the control and heat shock-treated groups were cultured in 24-well plates (0.8 × 10^3^ cells per well) and fixed with 4% paraformaldehyde at room temperature for 30 min. The cells were treated with 0.5% Triton X-100 at room temperature for 15 min and washed three times with PBS at room temperature for 5 min each time. Then, the cells were incubated with 10% goat serum at room temperature for 40 min. Then, the cells were incubated with polyclonal rabbit anti-GDNF receptorα-1 (GFRα1) (1:100; sc-10716, Santa Cruz Biotechnology, Santa Cruz, CA, USA) or polyclonal mouse anti-Promyelocytic Leukaemia Zinc Finger (PLZF) (1:150, sc-22839, Santa Cruz Biotechnology) primary antibodies at 4 °C overnight. Finally, the cells were incubated with FITC- or TRITC-conjugated secondary antibodies (1:200, A22120-1, Abbkine, Wuhan, Chian) at 37 °C for 30 min and stained with DAPI for 5 min. We stained at least three sections for each group, and images were evaluated in at least three randomly selected fields per section under a magnification of × 400 with an inverted fluorescence microscope (IX53, Olympus, Tokyo, Japan).

### Apoptosis detection

SSCs from the control group and the heat shock-treated groups (43 °C, 45 min or 60 min) were cultured in 6-well plates at a density of 5 × 10^5^ cells/well. Two and 18 h after the heat shock treatment, the cells were processed using an Annexin V-FITC Apoptosis Assay Kit (BB-4101-2, Bestbio, China) according to the manufacturer’s instructions. Briefly, cells were harvested, washed with 1 ml PBS and resuspended in 100 μl staining buffer for 15 min at room temperature. After centrifugation, the staining buffer was aspirated, and cells were resuspended in 100 μl PBS for analysis. Samples were analyzed with the flow cytometer (BD Accuri™ C6, BD Biosciences). The 488-nm laser was used for excitation. Debris and doublets were gated out. At least 10,000 events of single cells per sample were collected. Additional single-labelled samples were prepared, which contain dead cells and serve as a positive control for single staining of Annexin V or PI, respectively. BD Accuri™ C6 Software was used to analyse the data. We also used ATUNEL BrightGreen Apoptosis Detection Kit (A211-01, Vazyme, Nanjing, China) to stain the apoptotic SSCs according to the manufacturer’s protocol. Images were obtained with an inverted fluorescence microscope (IX53, Olympus).

### RNA-Seq and data analysis

Total RNA was extracted from SSCs 2 h after the heat shock treatment using TRIzol Reagent (15596-018, Life Technologies, Carlsbad, CA, USA) following the manufacturer’s instructions, and the RIN numbers were assessed to inspect RNA integrity with an Agilent Bioanalyzer 2100 (Agilent Technologies, Santa Clara, CA, USA). Qualified total RNA was further purified with an RNAClean XP Kit (A63987, Beckman Coulter, Inc., Kraemer Boulevard Brea, CA, USA) and an RNase-Free DNase Set (79254, Qiagen, GmBH, Germany). Total RNA (1 μg) was also extracted from SSCs 2 h after the heat shock treatment and used for library preparation according to Illumina standard instructions (TruSeq Stranded RNA LT Guide). An Agilent 2100 Bioanalyzer was employed to evaluate the concentration and size distribution of the cDNA library before sequencing with an Illumina HiSeq. The protocol for high-throughput sequencing was fully performed in strict accordance with the manufacturer’s instructions (Illumina). The raw reads were filtered with Seqtk before being mapped to the genome using TopHat (version: 2.0.9) [20]. The fragments of genes were counted using HTSeq followed by trimmed mean of M value (TMM) normalization [21, 22]. Significant differentially expressed genes (DEGs) were identified as those determined to have a false discovery rate (FDR) passing the threshold (*Q* < 0.05) and a fold change > 2 using edgeR software [23].

### Quantitative real-time PCR

Some differentially expressed genes were validated by quantitative real-time (qRT) PCR. The primers were synthesized as shown in Table 1. We used the Tip Green qPCR SuperMix (AQ141, TransGen Biotech, Beijing, China) in a 20-μl reaction volume on a 7500 Fast Real-Time PCR System, and the reaction conditions were set at 94 °C for 30 s followed by 43 cycles of 94 °C for 5 s and 60 °C for 34 s. The qRT-PCR primers were synthesized by Sangon Biotech (Shanghai) Co, Ltd. The data analysis was performed using the 2^−△△CT^ method.

**Table 1:** Primers used for qRT-PCR

Gene	Forward primer sequence (5′-3′)	Reverse primer sequence(5′-3′)	Product length (bp)
Oncostatin M (Osm)	ACTTCCTCCTTTCCCTGTGG	CACCCAGAGGTCCAGGTATC	101
Socs3	GTACTGAGCCGACCTCTCTC	ATCCAGGAACTCCCGAATGG	129
Il6ra	TGCTCTGCTTCAGGGAATGA	AGGCCACTCAGTCAAACGTA	121
Il13	GGTTCTGTGTAGCCCTGGAT	GGTTACAGAGGCCATGCAAT	90

### Western blot

The in vitro-cultured SSCs were collected, and protein lysates (keygentec, Nangjing, China) were extracted at 4 °C for 30 min. The proteins were denatured in 5× SDS loading buffer at 100 °C for 5 min. The total cell proteins were resolved by 12% SDS-PAGE and transferred onto PVDF membranes. After blocking with 5% nonfat milk powder in TBST for 1 h, the PVDF membranes were incubated with polyclonal rabbit anti-Osm (1:1000, A6163, ABclonal, Woburn, MA, USA), monoclonal mouse anti-β actin (1:1000, sc-58673, Santa Cruz Biotechnology), polyclonal rabbit anti-Socs3 (1:1000, 14025-1-AP, Proteintech, Wuhan, China), monoclonal rabbit anti-p-Stat3 (1:1000, ab76315, Abcam), polyclonal rabbit anti-p-Akt (1:1000, AF0016, AFFinify) and polyclonal rabbit anti-GAPDH (1:1000, bs2188R, Bioss, Beijing, China) primary antibodies at 4 °C overnight. Then, horseradish peroxidase-conjugated goat anti-mouse or anti-rabbit(1:20000, A21020-1, Abbkine) secondary antibodies were used at room temperature for 1 h, and the protein expression was detected by enhanced chemiluminescence (RM00021, ABclonal); gel imaging was performed with a ChemiDoc™ XRS+ (Bio-Rad, USA) with Image Lab™ software, and gray value analysis was performed with ImageJ.

### Cell cycle analysis

SSCs from the control group and the heat shock-treated group (43 °C, 45 min) were seeded in 6-well plates at the density of 2 × 10^5^ cells/well. Two hours and 18 h after the heat shock treatment, the cells were trypsinized, collected and washed with PBS before 70% freezer-chilled ethanol was use to fix the cells for 2 h at 4 °C. A cell cycle kit (KGA512, KeyGEN) was used to detect the SSC cycle according to the manufacturer’s instructions. Briefly, cells resuspended in 500 μl of propidium iodide buffer containing propidium iodide and ribonuclease A (9:1) were incubated in the dark for 40 min. The intensity of fluorescence staining in the cells was measured using a flow cytometer (BD Accuri™ C6, BD Biosciences, USA). Debris and doublets were gated out. The particular phase of the cell cycle with DNA content in G0/G1, S and G2/M was estimated using BD Accuri™ C 6 software.

### JAK-STAT signalling pathway inhibition

SSCs were plated at a density of 2 × 10^3^ cells/well in 96-well plates, cultured for 2 h, washed with medium and then cultured for an additional 20 h in SSC medium in the presence or absence of 1.25 μM, 2.5 μM or 5 μM WP1066 (A JAK-STAT signalling pathway inhibitor; A4140, APExBIO). SSC viability was detected with CCK-8 kit 0 h, 4 h, 8 h, 12 h, 16 h and 20 h after WP1066 treatment.

### Statistical analysis

The dates are presented as the mean ± SD, and three independent experiments were repeated at least. The data were analysed using one-way ANOVA in SAS software (SAS Institute Inc., Cary, NC, USA). *P* < 0.05 was considered to indicate a statistically significant difference, and *P* < 0.01 was considered to indicate a highly significant difference among the different treatment groups.

## Results

### Heat shock treatment inhibits SSC proliferation

**Fig. 1:**
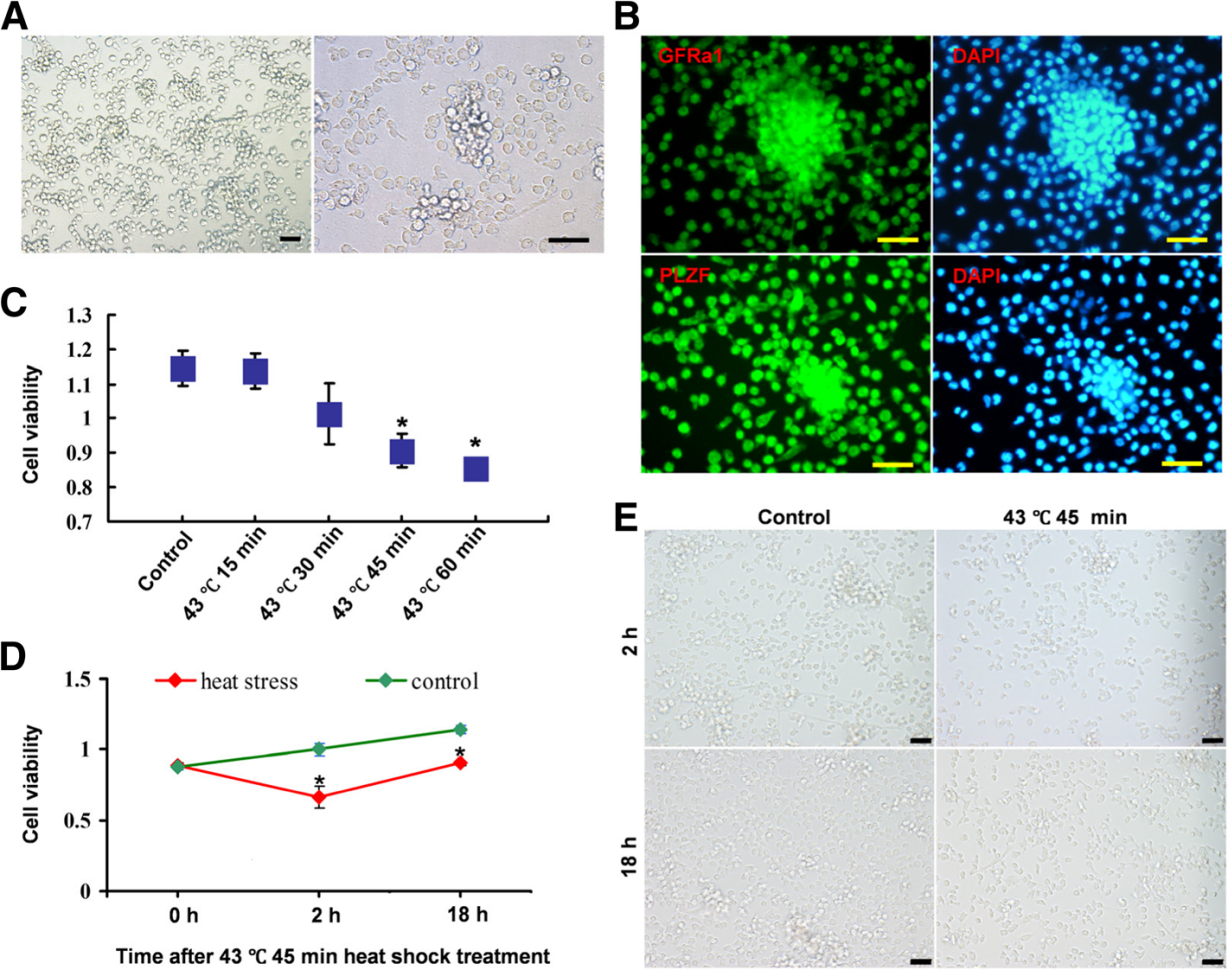
Heat shock treatment inhibits SSC proliferation. **a** The CD1 SSC line can be stably cultured in vitro. **b** The SSC surface marker proteins GFRα-1 and PLZF were expressed in in vitro-cultured SSCs. **c** Viability of SSCs 18 h after heat shock treatment. Heat shock treatment at 43 °C for 45 min or at 43 °C for 60 min significantly inhibited the proliferation of SSCs 18 h after treatment. **d** The viability of SSCs was significantly reduced as early as 2 h after 45 min of 43 °C heat shock treatment. **e** Two and 18 h after treatment, compared with the control group, the cell density in the 43 °C 45-min heat shock-treated group was significantly reduced. *P < 0.05 compared with the control group. Bar = 50 μm

The CD1 SSC line could be stably cultured in vitro in our laboratory (Fig. 1a). GFRα-1 and PLZF are mostly used as consensus markers for SSC identification in rodents [24–26]. Our results also demonstrated that this cell line expressed the SSC surface marker proteins GFRα-1 and PLZF during in vitro culture (Fig. 1b). To explore the effects of heat stress on SSCs, we treated them at 43 °C for 10 min, 15 min, 30 min, 45 min and 60 min. We found that the heat shock treatment at 43 °C for 10 min, 15 min or 30 min had no significant effects on the proliferation of SSCs 18 h after treatment. However, when the treatment time was extended to 45 min or 60 min, proliferation was significantly inhibited (*P* < 0.05) (Fig. 1c). To further clarify the early dynamic changes in the proliferation after heat shock treatment, the viability of in vitro-cultured SSCs was measured at 0 h, 2 h and 18 h after 45 min of 43 °C heat shock treatment. We found that although the proliferative activity remained stable before the treatment, proliferation was significantly reduced as early as 2 h after the treatment (Fig. 1d). Although there was no significant difference in cell morphology 2 h after 45 min of 43 °C heat shock treatment, cell density was significantly reduced. Eighteen hours after treatment, the number of cells in 45 min of 43 °C heat shock-treated group was recovered but was still significantly lower than that in the control group (Fig. 1e). These results indicated that the regulatory mechanism of SSC regeneration was changed 2 h after the heat shock treatment.

### Heat shock treatment did not cause SSCs to undergo apoptosis

**Fig. 2:**
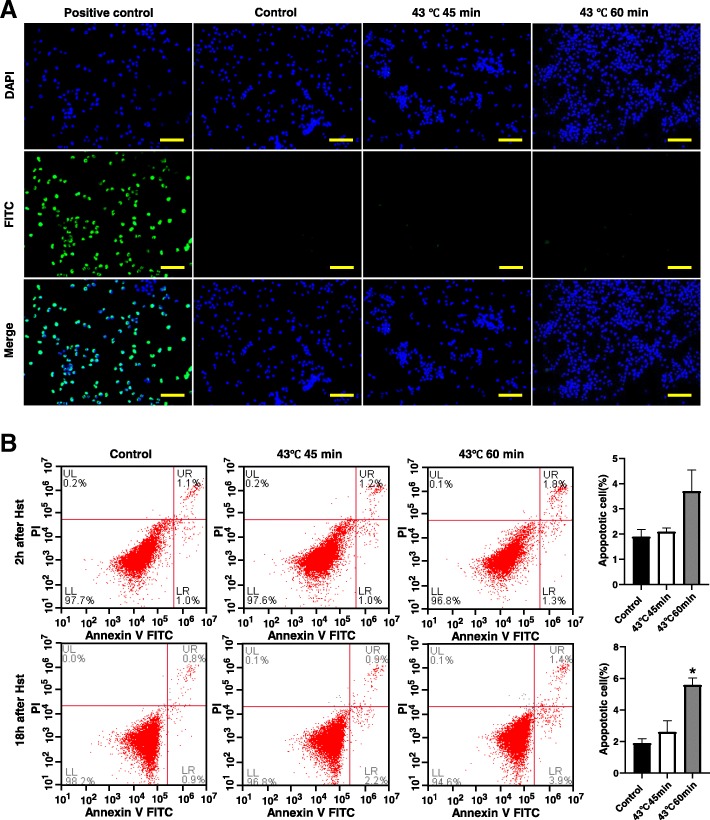
Heat shock treatment with at 43 °C for 45 min did not cause in vitro-cultured SSCs to undergo apoptosis. **a** The TUNEL test results showed that apoptosis of SSCs was not increased 2 h after heat shock at 43 °C for 45 min or at 43 °C for 60 min. **b** The Annexin V test results showed that 45 min of 43 °C heat shock treatment did not cause increased apoptosis of SSCs 2 h and 18 h after treatment, while 60 min of 43 °C heat shock treatment led to increased apoptosis of SSCs 18 h after heat shock treatment. Bar = 200 μm

To further clarify whether the proliferation inhibition of SSCs was caused by apoptosis, we investigated the apoptosis of SSCs after heat shock treatment. Our TUNEL test results showed that treatment at 43 °C for 45 min or 43 °C for 60 min did not cause increased apoptosis of SSCs 2 h after the heat shock treatment. The numbers of FITC-positive SSCs in the groups treated for 45 min and 60 min at 43 °C were similar to that in the control group 2 h after the heat shock treatment (Fig. 2a). The Annexin V test results showed that treatment at 43 °C for 45 min did not cause increased apoptosis of SSC. The percentages of apoptotic cells in the group at 43 °C for 45 min and the group treated at 43 °C for 60 min were 2.1 ± 0.14% and 3.7 ± 0.85% 2 h after the heat shock treatment, respectively; these values were not significantly different from the value in the control group. Eighteen hours after the heat shock treatment, the SSC apoptosis rate in the group treated at 43 °C for 45 min (2.6 ± 0.71%) remained unchanged, while the SSC apoptosis rate in the group treated at 43 °C for 60 min (5.6 ± 0.42%) was significantly increased (Fig. 2b). Therefore, in subsequent studies, we treat the in vitro-cultured SSCs at 43 °C for 45 min and analysed the transcriptome differences between the heat shock-treated group and the control group.

### Analysis of differentially expressed genes after heat shock treatment

**Fig. 3:**
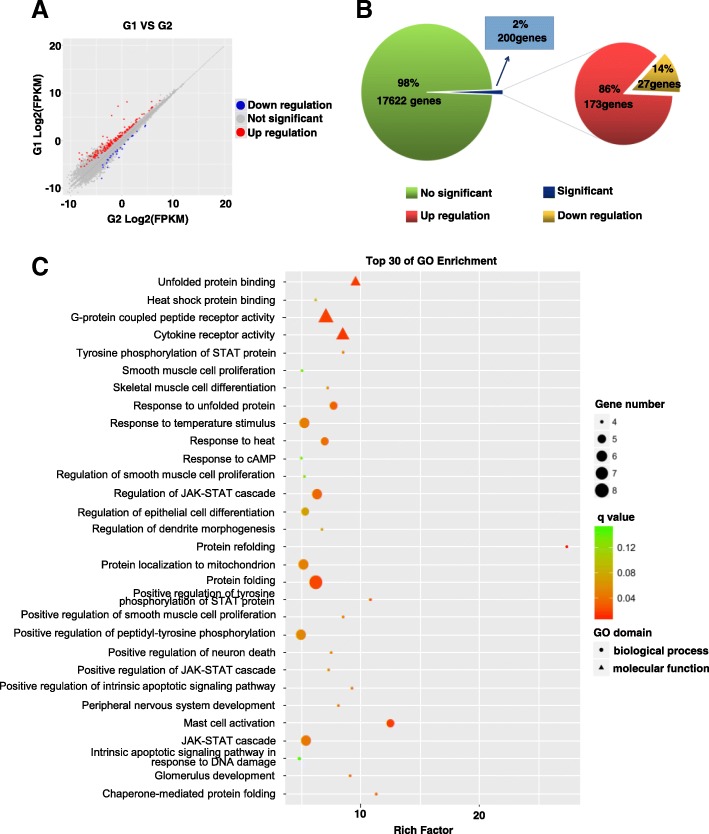
Analysis of the differentially expressed genes after heat shock treatment. **a** Scatter diagram of gene expression in the heat shock-treated group. Red indicates upregulated genes, and blue indicates downregulated genes. **b** Pie chart representation of the percentages of genes that were significantly upregulated and downregulated in the control and treated groups. **c** Top 30 enrichment GO terms obtained through GO analysis of the differentially expressed genes

To explore the gene expression of heat shock-treated SSCs, we performed RNA-Seq analysis on in vitro-cultured SSCs 2 h after 45 min of 43 °C heat shock treatment. In total, 17,822 genes were identified from the heat shock-treated group and control group. We found that the expression of 200 genes changed significantly (with at least a twofold difference between the two groups and an FDR less than 0.05) after the heat shock treatment, among which 173 were upregulated and 27 were downregulated in the heat shock-treated group (Fig. 3).

### Gene Ontology analysis of the differentially expressed genes

Gene Ontology (GO) analysis was used to characterize the functions of the DEGs obtained from RNA-Seq. GO terms can be divided into three categories: the molecular function, biological process and cellular component categories. According to the selected differentially expressed genes, the hypergeometric distributions between the differentially expressed genes and certain (several) specific GO classification terms are calculated. GO analysis will return a *P* value for each GO term associated with the differentially expressed genes. The top 30 enriched terms are shown in Fig. 3c. The selected top 10 GO enrichment terms were protein folding (e.g. *Dnajb1*, *Dnaja1*), chaperone-mediated protein folding (e.g. *Clu*, *Hsph1*), positive regulation of tyrosine phosphorylation of STAT protein (e.g. *Hes1*, *Osm*), unfolded protein binding (e.g. *Serpinh1*, *Dnajb1*), positive regulation of intrinsic apoptotic signalling pathway (e.g. *Bbc3*, *Skil*), tyrosine phosphorylation of STAT protein (e.g. *Il6ra*, *Il13*), cytokine receptor activity (e.g. *Gfra2*, *Il18r1*), response to unfolded protein (e.g. *Hsph1*, *Crebrf*), response to heat (e.g. *Hsp90aa1*, *Osm*) and regulation of JAK-STAT cascade (e.g. *Gfra2*, *Socs3*) (Table 2).

### Kyoto Encyclopedia of Genes and Genomes analysis of the differentially expressed genes

**Fig. 4:**
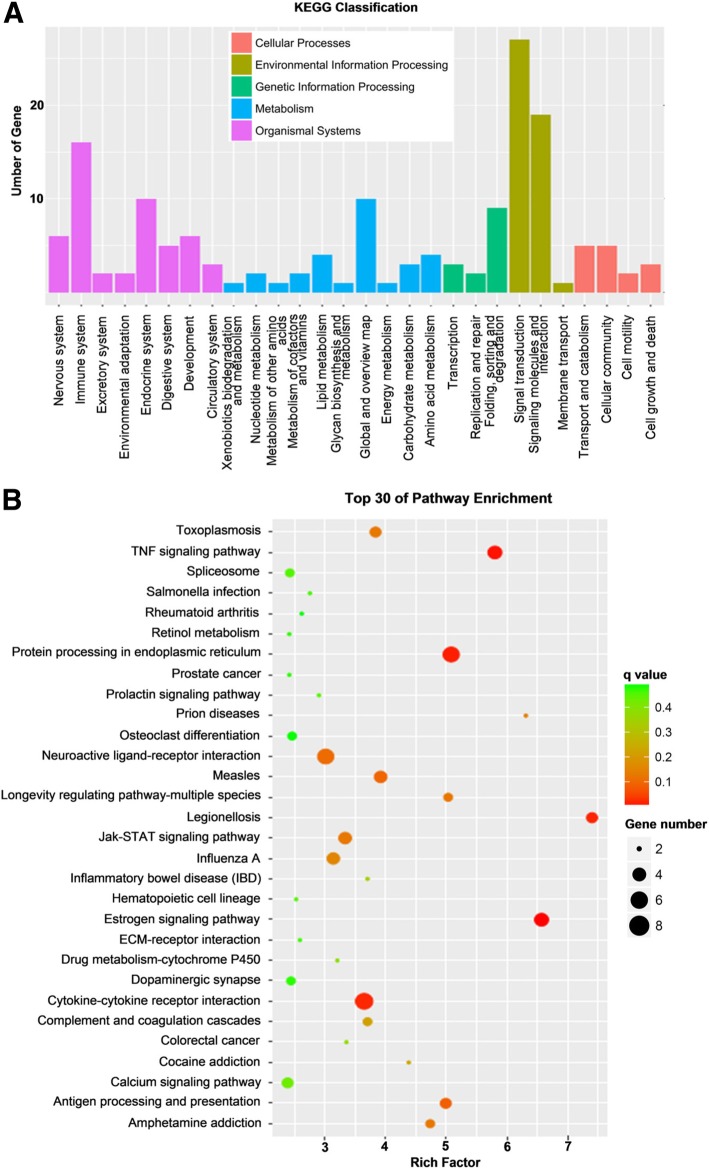
KEGG pathway analysis of the differentially expressed genes (DEGs). **a** KEGG classification. **b** Top 30 enriched pathways

The Kyoto Encyclopedia of Genes and Genomes (KEGG) is a database for systematic analysis of gene function and genome information. KEGG enrichment analysis of differentially expressed genes can reveal pathways with significant enrichment, which is helpful for finding significantly altered biological regulatory pathways. To further explore the roles of the DEGs in SSC proliferation after heat shock treatment, we tested whether the DEGs were enriched in certain KEGG pathways. The statistical results showed that the number of DEGs in environmental information processing pathways was 37, which was the largest number. Twenty-seven of the 37 genes were involved in the signal transduction pathway, and 19 were associated with signalling molecules and interaction pathways. The numbers of DEGs associated with organismal systems pathways, metabolism, cellular processes and genetic information processing were 32, 14, 13 and 11, respectively (Fig. 4a). The top 30 signalling pathways that were enriched in the heat shock-treated group versus the control group are shown in Fig. 4b. Moreover, we screened 10 signalling pathways on the basis of our research purposes and cell phenotypes. These DEGs were significantly enriched for the estrogen signalling pathway, the TNF signalling pathway, protein processing in the endoplasmic reticulum, cytokine-cytokine receptor interaction, the JAK-STAT signalling pathway, ECM-receptor interaction, spliceosomes, apoptosis, the PI3K-Akt signalling pathway and MAPK the signalling pathway (Table 3).

**Table 2:** Selected top 10 of GO enrichment

GO-ID	GO term	Type	Up-genes	Down-genes	*P* value
GO:0042026	Protein refolding	Biological process	Dnajb1, Dnaja1, Hspa1l, Hsp90aa1	–	1.96E−06
GO:0061077	Chaperone-mediated protein folding	Biological process	Clu, Hsph1, Dnajb1, Unc45b	–	9.11E−05
GO:0042531	Positive regulation of tyrosine phosphorylation of STAT protein	Biological process	Hes1, Osm, Il6ra, Il13	–	0.00011
GO:0051082	Unfolded protein binding	Molecular function	Serpinh1, Dnajb1, Hsp90aa1, Dnaja1, Hspa1l	–	4.71E−05
GO:2001244	Positive regulation of intrinsic apoptotic signaling pathway	Biological process	Bbc3, Skil, Clu, Bcl2l11	–	0.00022
GO:0007260	Tyrosine phosphorylation of STAT protein	Biological process	Il6ra, Il13, Osm, Hes1	–	0.00032
GO:0004896	Cytokine receptor activity	Molecular function	Gfra2, Il18r1, Ccr4, Il1rl1, Ccr9, Il6ra	–	2.44E−05
GO:0006986	Response to unfolded protein	Biological process	Hsph1, Crebrf, Hsp90aa1, Hspa4l	Chac1	0.00014
GO:0009408	Response to heat	Biological process	Hsp90aa1, Osm, Socs3, Trp53inp1, Dnaja1	–	0.00024
GO:0046425	Regulation of JAK-STAT cascade	Biological process	Gfra2, Socs3, Il13, Il6ra, Osm, Hes1	–	0.00014

### JAK-STAT signalling pathway and S phase cell cycle arrest of SSCs

**Fig. 5:**
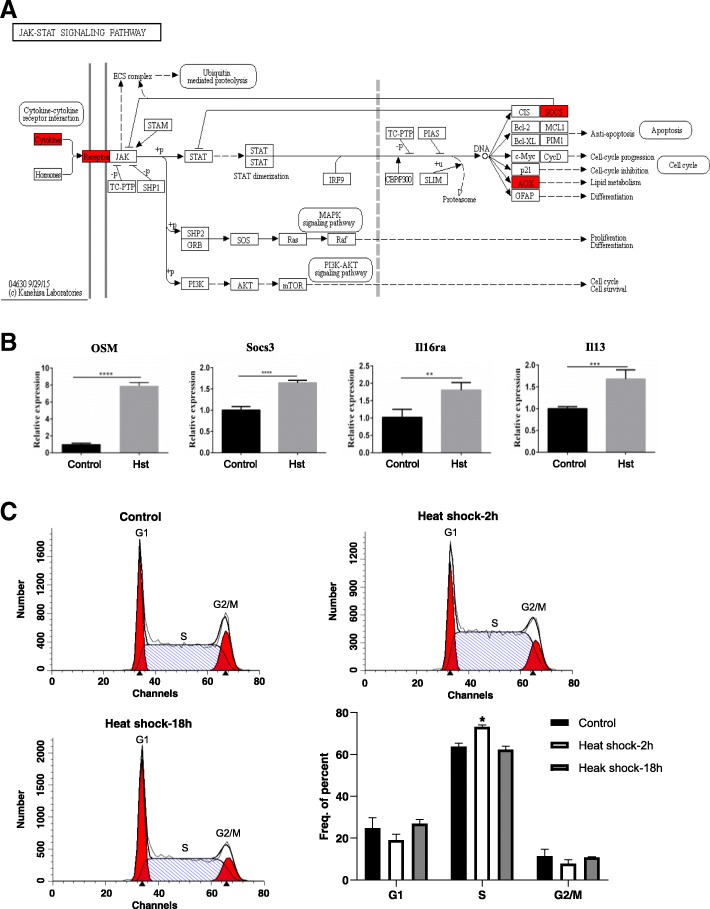
Heat shock treatment-induced SSC cell cycle inhibition. **a** The KEGG JAK-STAT signalling pathway responds to cell cycle progression and inhibition; the genes highlighted in red were enriched and upregulated. **b** Validation of the enriched and upregulated genes in the Jak-Stat signalling pathway by quantitative PCR. **c** Heat shock treatment-mediated S phase cell cycle arrest in in vitro-cultured SSCs 2 h after heat shock treatment. However, 18 h after heat shock treatment, S phase cycle arrest was eliminated, and the proportion of cells in S phase returned to a normal level

To verify the results of RNA-Seq in the JAK-STAT signalling pathway, we selected DEGs in the JAK-STAT signalling pathway (Fig. 5a) and validated the gene expression levels by qRT-PCR. We found that the Osm, Socs3, Il6ra and Il13 genes were significantly upregulated (Fig. 5b). Upregulation of these genes in the JAK-STAT signalling pathway may prevent cell cycle progression. Therefore, we studied the changes in the cell cycle in SSCs 2 h and 18 h after 45 min of 43 °C heat shock treatment. The results showed that the proportion of cells in the S stage increased from 63.79 ± 1.59% before treatment to 73.19 ± 0.92% 2 h after treatment. Eighteen hours after the heat shock treatment, there was no significant difference in the S phase cell proportion between the group treated at 43 °C for 45 min (62.32 ± 1.68%) and the control group (Fig. 5c). These results indicated that the SSCs showed short-term S phase arrest after the heat shock treatment.

**Fig. 6:**
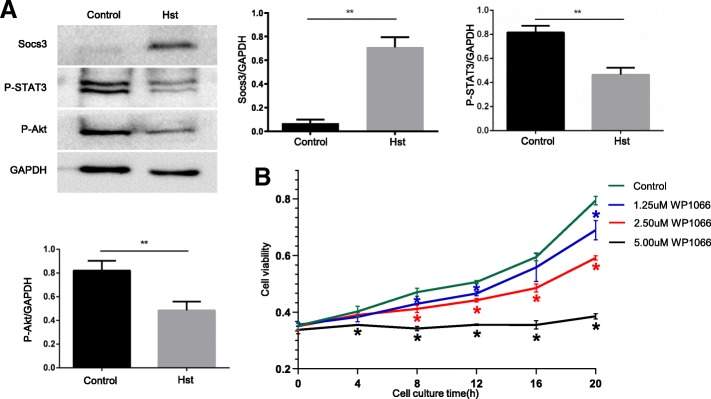
The JAK-STAT signalling pathway is involved in the inhibition of spermatogonial stem cell regeneration induced by high temperature. **a** The expression of the negative feedback inhibitor Socs3 in the JAK-STAT signalling pathway was upregulated 2 h after heat shock treatment, while the phosphorylation of STAT3 was reduced. The phosphorylation of Akt in the PI3K-Akt signalling pathway, which was regulated by the JAK-STAT signalling pathway, was found to be reduced. **b** Inhibition of the JAK/STAT signalling pathway by WP1066 in non-treated SSCs induced a decrease in proliferation. The asterisk indicates a significant difference from the control group

To further clarify whether the JAK-STAT pathway was involved in the inhibition of SSC self-renewal caused by heat shock treatment, we detected the expression and phosphorylation of some proteins in the JAK-STAT pathway. We found that the expression of Socs3 protein increased and the phosphorylation of STAT3 decreased after the heat shock treatment(Fig. 6a), indicating that the JAK-STAT pathway was inhibited. Our bioinformatics analysis results showed that the PI3K/Akt pathway was the downstream signalling pathway of the JAK-STAT pathway in this study(Fig. 5a), and our experimental results also showed that the phosphorylation of Akt decreased after the heat shock treatment (Fig. 6a). To further verify the function of the JAK-STAT pathway, we blocked the JAK-STAT pathway with the inhibitor WP1066 in in vitro-cultured SSCs and found that SSC self-renewal was inhibited (Fig. 6a), indicating that the JAK-STAT pathway promotes SSC self-renewal. Based on these studies, we found that the JAK-STAT pathway is involved in the inhibition of SSC self-renewal after heat shock treatment.

## Discussion

In this study, for the first time, we studied the developmental potential and overall gene expression patterns of in vitro-cultured SSCs after heat shock treatment. Previous research has shown that heat shock treatment induces adverse effects in the testes of mice, rats and cows, including germ cell apoptosis, low-quality sperm and abnormal DNA and chromatin structures [27–29]. However, the effects of heat shock treatment on SSC development were not fully understood. In this study, we found that high temperature suppressed SSC self-renewal through S phase cell cycle arrest but not apoptosis. These results provide new insights for the diagnosis and treatment of human asthenospermia associated with high temperature.

We conducted experiments on the effects of high temperature on the development of the in vitro-cultured SSC cell line CD-1. The main limitation of in vivo SSC research is that the number of SSCs in vivo is very small. Usually, SSCs account for as few as 0.03% of all adult testis cells [19]. It is thus difficult to detect the influence of high temperature on SSC development and gene expression in vivo. However, the use of SSC cell lines in this study overcame this limitation. The CD-1 SSC cell line cultured in our laboratory stably expressed PLZF and Gfra-1 protein. PLZF and Gfra-1 are mostly used as consensus markers for SSC identification in rodents [30]. PLZF, a transcription-inhibiting protein, is expressed in the nuclei of A single (As), A paired (Apr) and A aligned (Aal) spermatogonia and is an essential autocrine signalling protein for SSC self-renewal [31]. GFRa1 is a receptor of glial cell line-derived neurotrophic factor (GDNF). GFRa1 is expressed in As, Apr and Aal4 spermatogonia in mice [26]. Thus, in this study, we used the CD-1 SSC line to detect the effects of heat shock treatment on SSC proliferation.

We established a stable heat shock treatment scheme for in vitro-cultured SSCs. In our previous experiment, we treated the testes with a single heat shock treatment in a hot water bath at 43 °C for 15 min [9, 32]. Therefore, in this study, we treated SSCs cultured in vitro at 43 °C in a CO_2_ incubator for different times, including 10 min, 15 min, 30 min, 45 min and 60 min. We found that heat shock treatment at 43 °C for 45 min or 60 min significantly inhibited the proliferation of SSCs cultured in vitro. However, it remains unclear whether the SSC proliferation inhibition was caused by the inhibition of stem cell self-renewal or by apoptosis. Some researchers have found that 15 min of testicular heat shock treatment at 43 °C can induce differentiated germ cell apoptosis [33, 34]. Therefore, in this study, we also studied the effect of heat shock treatment on in vitro-cultured SSC apoptosis. Our results showed that treatment at 43 °C for 45 min or at 43 °C for 60 min did not cause increased apoptosis of SSCs, indicating that the inhibition of SSC proliferation was caused by the inhibition of SSC self-renewal rather than by apoptosis.

We performed RNA-Seq analysis of in vitro-cultured SSCs after the heat shock treatment and found 200 DEGs. The DEGs were screened by GO and KEGG analyses, and these genes were found to affect protein folding, protein localization and some types of cellular signalling. The RNA sequencing results showed that there were no GO terms associated with self-renewal or cell cycle arrest. However, we found that there were many GO terms related to the JAK-STAT signalling pathway. The JAK/STAT signalling pathway is a common pathway through which various cytokines and growth factors transmit signals in cells, mediating cell proliferation, differentiation, migration and apoptosis [35]. *Osm*, *Il6ra*, *Il13* and *Socs3* were enriched in the JAK/STAT signalling pathway, and these genes were upregulated significantly. *Osm* is a member of the IL-6 family and is a secretory factor and cell growth regulator. It has been reported in the literature that in A375 melanoma cells, *Osm* can upregulate P27 through a STAT1-dependent cellular pathway to inhibit cell proliferation [36]. In breast cancer, Osm can downregulate C-Myc and upregulate P21 and P53 through the JAK/STATs signalling pathway to inhibit the growth of cancer cells [37, 38]. Il6ra, a subunit encoding the interleukin 6 (IL6) receptor complex, is also a receptor subunit shared by other cytokines. Both IL-6 and Osm have been reported to inhibit MCF-7 cell growth in breast cancer [39]. Our bioinformatics analysis results showed that Socs3 was one of the upregulated genes; this gene can inhibit the JAK-STAT pathway and then the PI3K-Akt pathway through negative feedback. We confirmed that the expression of Socs3 protein was increased and that the phosphorylation of STAT3 was decreased in this study, indicating that SSC self-renewal may have been inhibited through the JAK-STAT pathway after heat shock treatment. It has been reported that Stat3 is not required for the self-renewal of spermatogonial stem cells but rather is needed for spermatogonial differentiation in mice [40]. However, the role of the JAK-STAT pathway in SSC self-renewal under stress conditions remains unclear [41]. In the Drosophila male germline, local activation of the Janus kinase-signal transducer and activator of transcription (Jak-STAT) pathway maintains stem cells, and germline stem cells lacking Jak-STAT signalling differentiate into spermatogonia without self-renewal [42], and JAK-STAT signalling regulation is also associated with chicken embryonic stem cell differentiation into male germ cells [43]. Our study showed that JAK/STAT signalling is also involved in this process during stress in in vitro-cultured SSCs.

**Table 3:** Selected top 10 of KEGG pathway enrichment

Pathway ID	KEGG pathway	Up-genes	Down-genes	*P* value
mmu04915	Estrogen signaling pathway	Hsp90aa1, Hspa1l, Fos, Hspa1a, Creb5, Hspa1b	–	1.29E−04
mmu04668	TNF signaling pathway	Fos, Creb5, Socs3, Cxcl3, Il18r1, Csf1	–	2.74E−04
mmu04141	Protein processing in endoplasmic reticulum	Hspa4l, Hsp90aa1, Hspa1l, Hsph1, Hspa1a, Dnaja1, Dnajb1, Hspa1b	–	1.24E−04
mmu04060	Cytokine-cytokine receptor interaction	Ccr9, Osm, Tnfrsf19, Inhbe, Ccr4, Il6ra, Il18r1, Il13, Csf1	–	7.87E−04
mmu04630	Jak-STAT signaling pathway	Osm, Aox1, Socs3, Il6ra, Il13	–	1.07E−02
mmu04512	ECM-receptor interaction	Itgb4, Sv2c	–	9.24E−02
mmu03040	Spliceosome	Hspa1l, Hspa1a, Hspa1b	–	8.08E−02
mmu04210	Apoptosis	Bcl2l11, Bbc3, Fos	–	9.22E−02
mmu04151	PI3K-Akt signaling pathway	Bcl2l11, Itgb4, Hsp90aa1, Osm, Creb5, Il6ra, Csf1	–	4.24E−02
mmu04010	MAPK signaling pathway	Hspa1l, Fos, Hspa1a, Hspa1b	–	1.96E−01

In addition, Osm was also enriched in the PI3K-AKt signalling pathway. In breast cancer, Osm can inhibit cancer cell growth by upregulating the P53 tumour suppressor gene. As a tumour suppressor gene, P53 functions to induce cell growth arrest, apoptosis, cell differentiation and DNA repair by the PI3K-AKt signalling pathway [44]. In our experiments, SSCs exhibited upregulation of the P53 signalling pathway-related genes *Bbc3*, *Trp53inp1* and *Trp53cor1* after heat stress. P53 can inhibit the proliferation of SSCs by activating the target genes *Bbc3*, *Trp53inp1* and *Trp53cor1* and inducing cell cycle arrest or apoptosis. Our findings suggest that the P53 signalling pathway may play an important role in the growth inhibition process of SSCs after high stress. In the PI3K/AKt signalling pathway, Akt can bind to MDM2 to phosphorylate its Ser166 and Ser186 sites and induce nuclear import or upregulate ubiquitin ligase activity, thereby promoting P53 inactivation or degradation and blocking P53-mediated transcription reaction [45, 46]. Our results showed that phosphorylated Akt expression was reduced in this study. Therefore, we hypothesized that *Osm* may act as a negative feedback regulator of the PI3K/AKt signalling pathway or induce a negative feedback regulator of the PI3K/AKt signalling pathway through the JAK/STAT signalling pathway, thereby mediating the inhibition of growth through the P53 signalling pathway after heat stress. Our results also showed a significant S phase arrest of SSCs after heat shock treatment. This result further indicates that the JAK/STAT and PI3K/AKt signalling pathways play important roles in SSC cycle arrest.

## Conclusion

Our results showed that heat shock treatment at 43 °C for 45 min significantly inhibited SSC proliferation through S phase cell cycle arrest but not apoptosis. We also screened some key genes involved in the cell proliferating signalling pathways by RNA-Seq. These results provide an important reference for subsequent study of the key genes in the molecular regulation mechanism of SSC self-renewal under heat shock treatment. At the same time, our findings also provide a reference for further study of the pathogenesis of male infertility caused by high temperature.

## Data Availability

Please contact the corresponding authors for data requests.

## Abbreviations

Aal: A aligned
Apr: A paired
As: A single
bFGF: Basic fibroblast growth factor
DAVID: Visualization and Integrated Discovery
DEGs: Differentially expressed genes
FBS: Foetal calf serum
GDNF: Glial cell line-derived neurotrophic factor
GO: Gene Ontology
Hst: Heat shock treatment
IL6: Interleukin 6
MAPK: Mitogen-activated protein kinase
Osm: Oncostain M
ROS: Reactive oxygen species
SSCs: Spermatogonial stem cells
